# Assessment of odor hedonic perception: the Sniffin’ sticks parosmia test (SSParoT)

**DOI:** 10.1038/s41598-020-74967-0

**Published:** 2020-10-22

**Authors:** David T. Liu, Antje Welge-Lüssen, Gerold Besser, Christian A. Mueller, Bertold Renner

**Affiliations:** 1 Department of Otorhinolaryngology, Head and Neck Surgery, Medical University of Vienna, Vienna, Austria; 2 Department of Otorhinolaryngology, University Hospital, University of Basel, Basel, Switzerland; 3 Institute of Experimental and Clinical Pharmacology and Toxicology, Friedrich-Alexander Universität Erlangen-Nürnberg, Erlangen, Germany; 4 Institute of Clinical Pharmacology, Medical Faculty Carl Gustav Carus, Technische Universität Dresden, Dresden, Germany

**Keywords:** Olfactory system, Diagnosis

## Abstract

Qualitative olfactory dysfunction is characterized as distorted odor perception and can have a profound effect on quality of life of affected individuals. Parosmia and phantosmia represent the two main subgroups of qualitative impairment and are currently diagnosed based on patient history only. We have developed a test method which measures qualitative olfactory function based on the odors of the Sniffin’ Sticks Identification subtest. The newly developed test is called Sniffin’ Sticks Parosmia Test (SSParoT). SSParoT uses hedonic estimates of two oppositely valenced odors (pleasant and unpleasant) to assess hedonic range (HR) and hedonic direction (HD), which represent qualitative olfactory perception. HR is defined as the perceivable hedonic distance between two oppositely valenced odors, while HD serves as an indicator for overall hedonic perception of odors. This multicenter study enrolled a total of 162 normosmic subjects in four consecutive experiments. Cluster analysis was used to group odors from the 16-item Sniffin’ Sticks Identification test and 24-additional odors into clusters with distinct hedonic properties. Eleven odor pairs were found to be suitable for estimation of HR and HD. Analysis showed agreement between test–retest sessions for all odor pairs. SSparoT might emerge as a valuable tool to assess qualitative olfactory function in health and disease.

## Introduction

The sense of smell enables us to interact with our environment and olfactory impairment results in a loss of critical information. Olfactory disorders (OD) can be classified into two major groups: Reduced perception of odor intensity (quantitative impairment) and distorted odor perception (qualitative impairment). The causes of such conditions are diverse, including head traumas, upper respiratory tract infections, neurodegenerative disorders, and OD secondary to sinonasal diseases. Olfactory (dys)function in neurodegenerative disorders such as Parkinson’s and Alzheimer’s disease (PD and AD) has been increasingly recognized as a potential biomarker in the assessment of onset or progression^1–3^. More importantly, previous studies also suggested that the major symptom of anhedonia (the inability to experience pleasure in commonly pleasant situations) in mood disorders might also be reflected at olfactory level, resulting in previously pleasant odors being perceived as less pleasant^4–6^.

Qualitative OD are generally divided into two main categories: Parosmia and phantosmia^7–9^. Parosmia is defined as a distorted odor perception in the presence of a triggering source^10, 11^. It is well known that patients usually experience the hedonic tone of these sensations as unpleasant (usually described as “rotten” or “foul”), which can either be odor-specific or non-specific triggered by any odor^8–10, 12, 13^. This is contrasted to a distorted odor perception in the absence of any odor source (phantosmia), which is also often described as unpleasant^8, 14, 15^. Both symptoms can occur alone or in combination with a reduced perception of odor intensities (quantitative OD). The prevalence of parosmia and phantosmia as stand-alone symptom were both estimated at ~ 4%^16, 17^. In patients with OD, a large variation in the prevalence of parosmia (between 13 and 32%) has been observed, probably revealing striking differences in how parosmia is assessed clinically^18–20^. Likewise, literature also remains contradictory on the prognostic value of parosmia in patients with quantitative OD, which emphasizes the necessity of further studies to elucidate the course of these symptoms^21–23^.

Testing of olfactory function has become indispensable for clinical counselling of patients with OD. This is mostly due to the fact that self-reports of olfactory function are unreliable on an individual level^24–28^. A widely used, reusable, and portable method for quantitative assessment of olfactory function is represented by Sniffin’ Sticks, which can be used to measure odor threshold, discrimination, and identification^29, 30^. A composite score (TDI) then allows to discriminate between normal (normosmia), reduced (hyposmia), or severely impaired (anosmia) olfactory function^31, 32^. The cut-off scores for these classifications are usually interpreted within predefined age groups, since olfactory function deteriorates with age^31–33^. Nevertheless, results—and associated cut-off scores—from young adults (olfactory reference group) serve as a general benchmark for olfactory function, since subjects from this age group demonstrate the best olfactory test results in quantitative terms^31^. Although objective methods are established in clinical routine for the assessment, diagnosis, and follow-up testing of quantitative OD, the diagnosis of qualitative OD is currently mainly based on the medical history or the use of a questionnaire only^27^. This shortcoming highlights the need for higher acceptance of previously proposed methods that focus on qualitative OD in a clinical context^34–39^.

Odor quality refers to the object (e.g., the smell of a rose) that is associated with the odor, whereas the hedonic feature of odors is defined as the valence (i.e., pleasant or unpleasant) of an odor^40, 41^. The perceived pleasantness of odors shows sex-specific differences and is strongly correlated with odor intensity^34, 42, 43^. Based on the consideration that olfactory threshold function is usually measured in patients that complain about reduced perception of odor intensity (quantitative OD), the main complaint of patients with parosmia or phantosmia (unpleasant hedonic perceptions) might serve as an objective parameter in qualitative OD. Such a method would be of clinical significance in counselling and follow-up of patients to elucidate factors that may modulate it^23^. Moreover, new insights into qualitative OD might also reveal the prognostic value of distorted odor perceptions in neurodegenerative diseases or even in mood disorders.

Therefore, built on the concept of pre-existing protocols, we developed a new test method which measures hedonic olfactory perception based on pairwise presented odors by Sniffin’ Sticks, that we called the Sniffin’ Sticks Parosmia Test (SSParoT). The aim of this study was to (i) define objective parameters that are exemplary for qualitative OD, (ii) evaluate the suitability of well-known odors for SSParoT testing, (iii) assess test–retest reliability of SSParoT, and (iv) present normative values of these newly defined parameters derived from normosmic subjects within the olfactory reference group.

## Results

### Developing SSParoT

In order to develop a method which measures qualitative olfactory dysfunction, we designed a diagnostic test method that utilizes odors that are hedonically oppositely valued. Based on the consideration that patients with parosmia and phantosmia usually complain about unpleasant odor perceptions, the SSParoT measures hedonic range (HR) and hedonic direction (HD) of pairwise presented, pleasant and unpleasant odors based on a 9-point hedonic scale (Supplementary Fig. S1). The HR represents the perceptible range, while the HD depicts the balance or in-balance between two hedonically oppositely valenced odors. Odors are presented pairwise using felt-tip pens starting with the pleasant odor (counterbalanced).

### Eight odors from the Sniffin’ Sticks Identification test are suitable for hedonic testing

To determine whether the 16 odors from the German version of the Sniffin’ Sticks Identification test are suitable for the SSParoT method, we applied these to our cohort of 50 normosmic subjects. Odors were presented according to the presentation order of the original 16-item Identification test and participants were asked to rate hedonic estimate and intensity during one visit. Descriptive statistics are reported, followed by cluster analysis to merge hedonically similar odors into three groups (i) pleasant, (ii) neutral, and (iii) unpleasant (Supplementary Fig. S2). In a final step, odors from the pleasant and unpleasant groups were paired with one another using two methods (method one: most pleasant odor with most unpleasant, method two: most pleasant odor with least unpleasant) and HR and HD were calculated for each pair. In order to depict the optimal pairing method, which was defined as equal HR between each pair of odors, we performed Kruskal–Wallis tests. We excluded rose (phenylethyl alcohol) from pairing, since this odor has shown to be perceptually unstable with high inter-individual variability during our investigation in suprathreshold testing and in threshold detection^45^.

**Figure 1:**
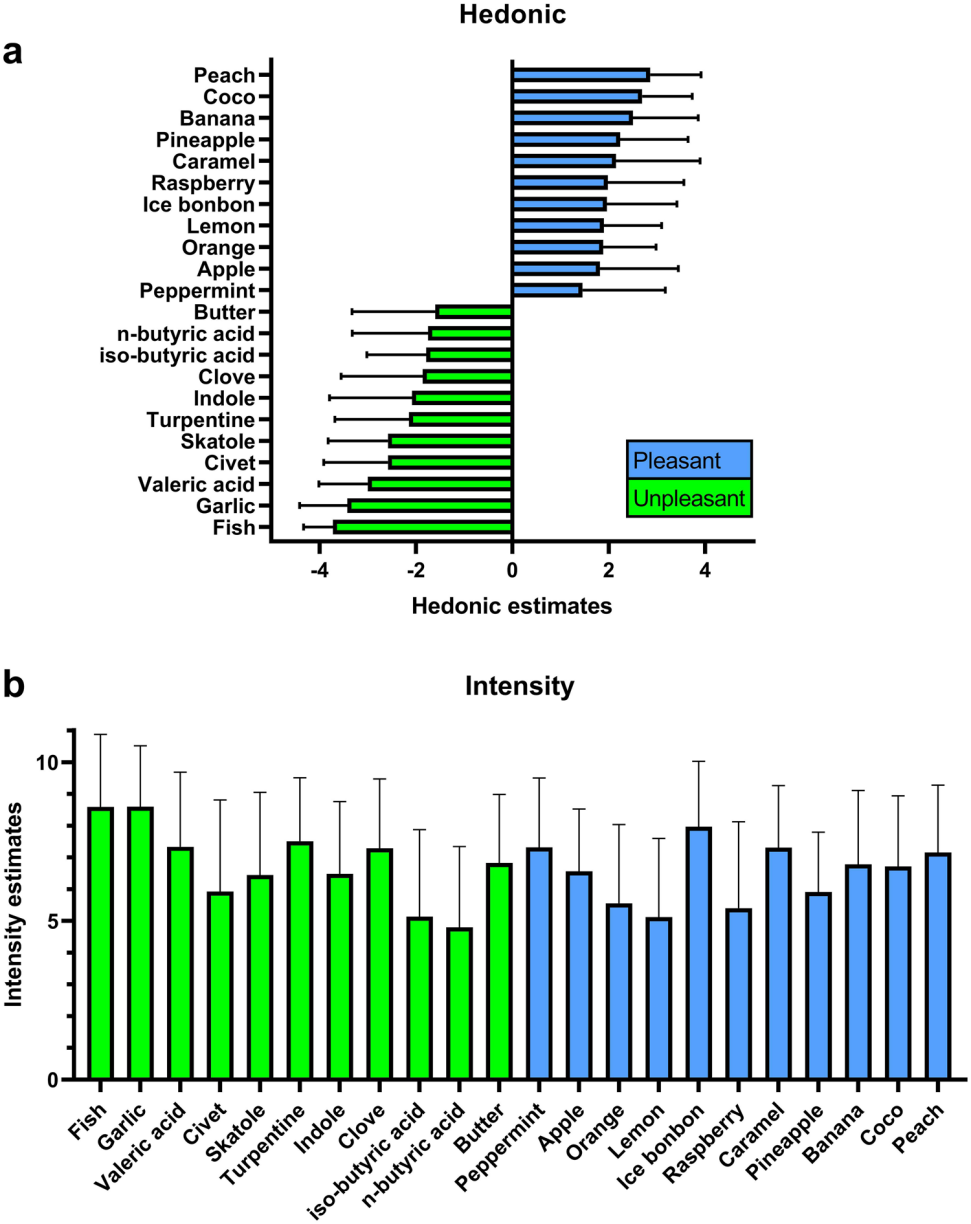
(**a**) Hedonic and (**b**) intensity ratings from odors used for the SSParoT. Columns are presented as mean ± standard deviation.

The results from hedonic and intensity rating for each of the 16 odors are detailed in Table 1 and Fig. 1. Hierarchical cluster analysis and dendrogram for hedonic ratings revealed a three-cluster solution for these odors to be optimal (Supplementary Figs. S2 and S3). According to the mean hedonic values, these were defined as: Cluster 1 (pleasant): Banana, Pineapple, (Rose), Apple, Peppermint, Cinnamon; Cluster 2 (neutral): Coffee, Shoe leather, Anise, Liquorice, Orange, Lemon; Cluster 3 (unpleasant): Fish, Garlic, Turpentine, Clove (Supplementary Table S1). Subsequent pairing using both methods resulted in 4 pairs for each method (Supplementary Table S2). Kruskal–Wallis tests for both methods revealed a significant effect of pair on HR in both models, Method 1: H = 10.32, p = 0.02, df = 3; method 2: H = 61, p < 0.01, df = 3. Since this difference was smaller using the first pairing method (most pleasant with least unpleasant), we chose this method for further analysis in order to benefit from homogenous HR values during the interpretation of results. Subsequently, odor pairs were defined as: Pair 1: Peppermint and Fish; pair 2: Apple and Garlic; pair 3: Pineapple and Turpentine; pair 4: Banana and Clove.

**Table 1:** Hedonic and intensity estimates from experiment 1 presented in clusters and stratified by sex (n = 50, 28 males, 22 female).

	Hedonic		Intensity	
	Mean (95% CI)		Mean (95% CI)	
	Male	Female	Male	Female
**Cluster 1**				
Banana	2.3 (1.7 to 2.9)	2.8 (2.3 to 3.2)	6.8 (5.8 to 7.7)	6.8 (5.8 to 7.8)
Pineapple	2.5 (2.0 to 3.0)	1.9 (1.3 to 2.6)	5.6 (4.9 to 6.3)	6.3 (5.5 to 7.1)
Rose	2.0 (1.1 to 2.8)	1.9 (0.8 to 3.0)	6.1 (5.1 to 7.1)	6.2 (5.3 to 7.1)
Apple	2.0 (1.4 to 2.6)	1.6 (0.8 to 2.4)	6.4 (5.5 to 7.3)	6.8 (6.2 to 7.4)
Peppermint	1.5 (0.8 to 2.2)	1.4 (0.6 to 2.2)	7.8 (6.9 to 8.6)	6.7 (5.8 to 7.6)
Cinnamon	0.6 (− 0.2 to 1.4)	0.5 (− 0.5 to 1.4)	5.5 (4.5 to 6.5)	5.5 (4.5 to 6.5)
**Cluster 2**				
Lemon	0.5 (− 0.2 to 1.3)	1.3 (0.7 to 1.9)	6.6 (5.8 to 7.4)	6.0 (5.0 to 6.9)
Orange	0.4 (− 0.3 to 1.1)	0.6 (− 0.2 to 1.3)	5.4 (4.6 to 6.1)	4.9 (3.9 to 5.9)
Liquorice	− 0.4 (− 0.9 to 0.2)	− 2.0 (− 2.7 to − 1.3)	5.2 (4.1 to 6.3)	5.1 (3.9 to 6.3)
Anise	− 0.4 (− 0.9 to 0.1)	0.1 (− 0.3 to 0.5)	3.6 (2.7 to 4.5)	4.3 (3.5 to 5.1)
Shoe leather	− 0.9 (− 1.4− to − 0.5)	− 0.5 (− 1.1 to 0.1)	5.4 (4.5 to 6.3)	4.8 (3.8 to 5.7)
Coffee	− 0.9 (− 1.7 to 0.0)	− 0.8 (− 1.7 to 0.2)	7.0 (6.1 to 7.9)	7.6 (6.8 to 8.4)
**Cluster 3**				
Clove	− 2.0 (− 2.6 to − 1.4)	− 1.6 (− 2.5 to − 0.8)	7.1 (6.2 to 8.0)	7.5 (6.6 to 8.4)
Turpentine	− 2.3 (− 2.9 to − 1.6)	− 2.0 (− 2.7 to − 1.3)	7.9 (7.2 to 8.5)	7.1 (6.0 to 8.1)
Garlic	− 3.4 (− 3.9 to − 3.0)	− 3.4 (− 3.8 to − 3.0)	8.1 (7.3 to 9.0)	9.2 (8.7 to 9.7)
Fish	− 3.9 (− 4.0 to − 3.7)	− 3.5 (− 3.9 to − 3.2)	8.5 (7.5 to 9.5)	8.7 (7.8 to 9.6)

*Cluster 1* pleasant, *Cluster 2* neutral, *Cluster 3* unpleasant.

Hence it seems that eight odors (merged into 4 pairs) of the 16-item Identification Sniffin’ Sticks were suitable for the assessment of HR and HD. In a next step, we assessed these odor pairs for test–retest reliability in another cohort of normosmic subjects.

### Agreement between test and retest measurements in HR and HD for odors selected from the Sniffin’ Sticks Identification test

The reproducibility in terms of test–retest reliability and agreement plays a significant role during the development process of new test methods. A new cohort of 33 subjects was therefore included in a test–retest study with at least one day in-between sessions (mean/SD = 12/7 days, min/max = 2/34 days). We applied the Bland–Altman statistical method, which compares two measurements (test–retest) of the same variable (i.e., HR and HD for each odor pair) to assess their degree of agreement. The interpretation of results is based on (i) the bias (mean difference between both sessions), (ii) the 95% Limits of Agreement (LoA, mean difference between both sessions ± 1.96 standard deviation of the difference between sessions), and (iii) the visual examination of Bland–Altman plots. A bias of zero would indicate no systematic differences between sessions, while a smaller 95% LoA indicates better agreement between two measurements. Subsequently, Wilcoxon matched-paired signed rank tests were performed to depict potential differences in HR and HD between both sessions.

**Figure 2:**
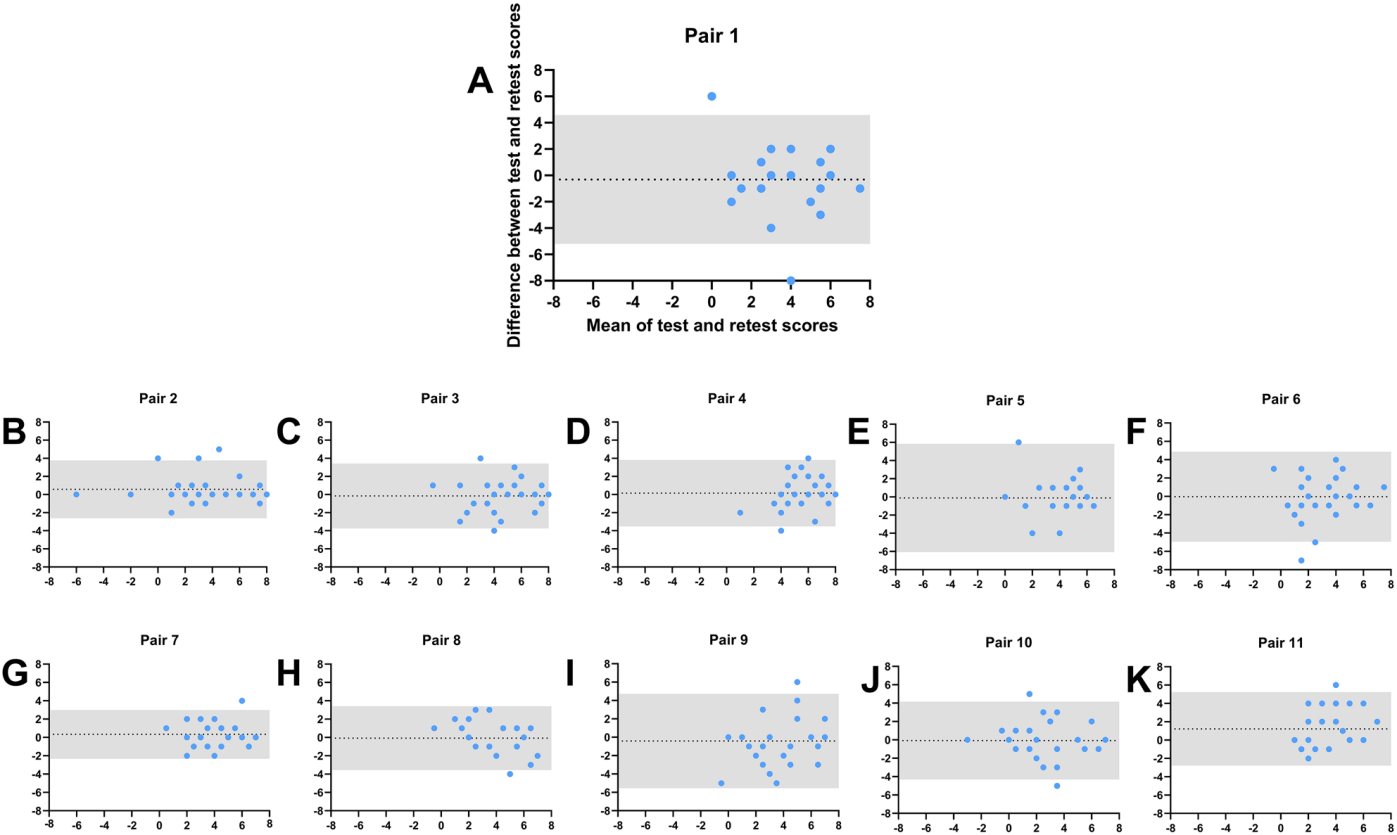
Bland–Altman plots of hedonic range (HR) for all 11 odor pairs. (**A**) Peppermint and Fish (**B**) Apple and Garlic (**C**) Pineapple and Turpentine (**D**) Banana and Clove (**E**) Peach and Butter; (**F**) Coco and n-Butyric acid (**G**) Caramel and iso-Butyric acid (**H**) Raspberry and Indole (**I**) Ice bonbon and Skatole (**J**) Lemon and Civet (**K**) Orange and Valeric acid. The dotted line represents the bias and 95% limits of agreement are indicated by the grey area.

**Figure 3:**
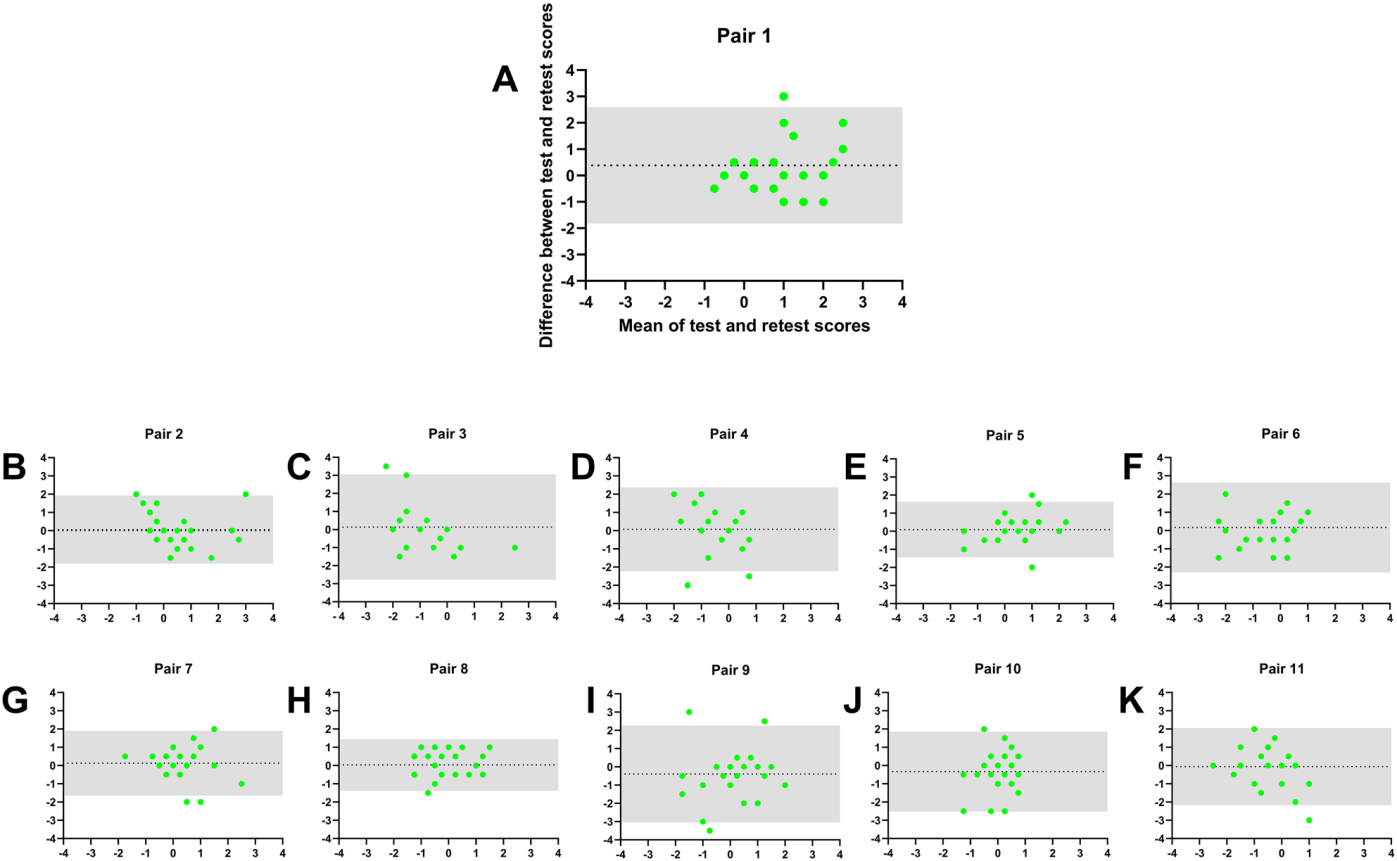
Bland–Altman plots of hedonic direction (HD) for all 11 odor pairs. (**A**) Peppermint and Fish (**B**) Apple and Garlic (**C**) Pineapple and Turpentine (**D**) Banana and Clove (**E**) Peach and Butter; (**F**) Coco and n-Butyric acid (**G**) Caramel and iso-Butyric acid (**H**) Raspberry and Indole (**I**) Ice bonbon and Skatole (**J**) Lemon and Civet (**K**) Orange and Valeric acid. The dotted line represents the bias and 95% limits of agreement are indicated by the grey area.

The bias of nearly zero for HR of all pairs: Pair 1 = − 0.31, pair 2 = 0.58, pair 3 = − 0.15, pair 4 = 0.15 indicated no systemic bias between both measurements. The 95% LoA were smaller for pair 2, 3, and 4 compared to pair 1, indicating higher variance between test–retest session for the latter (Fig. 2). Similarly, no systemic bias between test–retest sessions was found for HD: Pair  1 = 0.38, pair 2 = 0.06, pair 3 = 0.13, pair 4 = 0.08 (Fig. 3). Wilcoxon matched-paired signed rank tests revealed no differences in HD and HR between both sessions for pair 1–4 (all p > 0.05; Supplementary Table S3).

In a next step, we extended the currently used 8 odors from the 16-item Sniffin’ Sticks Identification test and evaluated 24 new odors based on felt-tip pens as carrier medium.

### Fourteen additional odors are suitable for hedonic testing

In order to optimize test performance in terms of higher accuracy, we evaluated 24 additional odors (Supplementary Table S4). Felt-tip pens were selected as carrier medium, since they have proved to be very suitable for hedonic testing during the first and second experiment. Concentrations of newly added odors were adjusted using the weakest and most intense odor from experiment one as benchmark. Hedonic and intensity estimates were documented in a new cohort of 52 subjects. Odors were paired using the method from the first experiment (most pleasant with least unpleasant). Olfactory performance was screened using the16-item Sniffin’ Sticks Identification test at the end of the third experiment.

Results from hedonic and intensity estimates are presented in Table 2 and Fig. 1. Following hierarchical cluster analysis revealed a three clusters solution to be optimal (Supplementary Figs. S4 and S5): Cluster 1 (pleasant): Peach, Coco, Caramel, Raspberry, Ice bonbon, Lemon, Orange, Cola, Apple, Strawberry, Pear, Menthol; Cluster 2 (neutral): Lavender, Grass, Ginger, Valerian, Cluster 3 (unpleasant): Valeric acid, Civet, Skatole, Indole, iso-Butyric acid, n-Butyric acid, Butter (Supplementary Table S5). Subsequent pairing using the same method as described above (see experiment 1; Supplementary Table S6) resulted in seven additional odor pairs: Pair 5: Peach and Butter; pair 6: Coco and n-Butyric acid; pair 7: Caramel and iso-Butyric acid; pair 8: Raspberry and Indole; pair 9: Ice bonbon and Skatole; pair 10: Lemon and Civet; pair 11: Orange and Valeric acid. Kruskal Wallis test revealed no significant effect of odor pair on HR (H = 5, p = 0.54, df = 3).

**Table 2:** Hedonic and intensity estimates of experiment III presented in clusters and stratified by sex (n = 52, 25 males, 27 females).

	Hedonic		Intensity	
	Mean (95% CI)		Mean (95% CI)	
	Male	Female	Male	Female
**Cluster 1**				
Peach	2.8 (2.4 to 3.2)	2.9 (2.5 to 3.3)	6.8 (5.9 to 7.9)	7.4 (6.7 to 8.2)
Coco	2.8 (2.4 to 3.3)	2.6 (2.2 to 2.9)	5.9 (5.0 to 6.8)	7.4 (6.7 to 8.2)
Caramel	2.0 (1.2 to 2.7)	2.3 (1.6 to 3.0)	7.0 (6.1 to 7.9)	7.6 (7.0 to 8.3)
Raspberry	1.7 (1.0 to 2.5)	2.2 (1.7 to 2.8)	5.2 (4.0 to 6.3)	5.6 (4.6 to 6.7)
Ice bonbon	2.3 (1.8 to 2.8)	1.7 (1.0 to 2.3)	7.7 (6.7 to 8.6)	8.2 (7.5 to 9.0)
Lemon	1.9 (1.4 to 2.3)	1.9 (1.4 to 2.4)	4.6 (3.7 to 5.5)	5.6 (4.6 to 6.7)
Orange	2.1 (1.7 to 2.6)	1.7 (1.3 to 2.1)	4.4 (3.5 to 5.3)	6.7 (5.8 to 7.6)
Cola	1.8 (1.1 to 2.4)	1.7 (1.1 to 2.3)	5.2 (4.3 to 6.2)	7.4 (6.7 to 8.2)
Apple	1.7 (1.1 to 2.3)	1.2 (0.5 to 1.9)	6.2 (5.3 to 7.1)	6.7 (6.0 to 7.4)
Strawberry	1.4 (0.8 to 2.0)	1.5 (0.8 to 2.2)	6.2 (5.3 to 7.0)	6.7 (6.0 to 7.4)
Pear	1.8(1.3 to 2.3)	1.1 (0.4 to 1.9)	5.6 (4.7 to 6.4)	7.1 (6.2 to 8.0)
Menthol	1.8 (1.1 to 2.4)	0.7 (0.0 to 1.4)	6.2 (5.3 to 7.1)	7.5 (6.5 to 8.5)
**Cluster 2**				
Lavender	1.5 (0.8 to 2.3)	0.8 (0.0 to 1.7)	7.2 (6.4 to 8.0)	7.0 (6.3 to 7.8)
Grass	0.2 (− 0.5 to 1.0)	0.6 (− 0.1 to 1.3)	4.4 (3.5 to 5.4)	6.1 (5.1 to 7.1)
Rose	0.2 (− 0.6 to 1.0)	0.3 (− 0.6 to 1.3)	4.6 (3.7 to 5.6)	7.3 (6.6 to 8.0)
Ginger	0.4 (− 0.3 to 1.2)	− 0.6 (− 1.3 to 0.0)	6.0 (5.0 to 6.9)	5.8 (5.1 to 6.5)
Valerian	0.1 (− 0.6 to 0.8)	− 0.6 (− 1.4 to 0.3)	6.4 (5.6 to 7.1)	5.5 (4.5 to 6.5)
**Cluster 3**				
Butter	− 1.6 (− 2.3 to − 0.8)	− 1.6 (− 2.3 to − 1.0)	6.7 (5.8 to 7.7)	6.9 (6.1 to 7.7)
n-Butyric acid	− 2.0 (− 2.6 to − 1.5)	− 2.0 (− 2.6 to − 1.5)	3.7 (2.8 to 4.6)	5.8 (4.8 to 6.8)
iso-Butyric acid	− 1.8 (− 2.3 to − 1.3)	− 1.8 (− 2.3 to 1.3)	4.0 (3.1 to 4.9)	6.1 (5.0 to 7.3)
Indole	− 2.0 (− 2.7 to − 1.3)	− 2.2 (− 2.9 to − 1.5)	6.5 (5.5 to 7.5)	6.4 (5.6 to 7.3)
Skatole	− 2.2 (− 2.8 to − 1.7)	− 2.9 (− 3.3 to − 2.5)	6.8 (5.6 to 7.9)	6.1 (5.2 to 7.1)
Civet	− 2.5 (− 3.2 to − 1.9)	− 2.6 (− 3.1 to − 2.2)	5.7 (4.5 to 6.9)	6.1 (5.0 to 7.3)
Valeric acid	− 3.0 (− 3.4 to − 2.5)	− 3.0 (− 3.4 to − 2.6)	6.5 (7.4 to 8.8)	8.1 (5.0 to 7.3)

*Cluster 1*  Pleasant, *Cluster 2* Neutral, *Cluster 3 * Unpleasant.

In a further step, we analyzed whether the newly defined odor pairs also show agreement between two measurements in an additional cohort of normosmic subjects.

### Agreement between test and retest measurements for HR and HD of additionally evaluated odors

To assess the reproducibility of HR and HD based on the seven newly depicted odor pairs, we conducted a test–retest reliability study (commensurate with experiment 2) with at least one day in-between sessions (mean/SD = 13/16 days, min/max = 1/56 days). We therefore included a new cohort of 27 subjects with no complaints regarding the sense of smell.

Calculation of the mean differences between test–retest sessions (retest minus first test-session) revealed a bias of nearly zero for all HR measures (Fig. 2): Pair 5–11 (bias) = 0.11; − 0.04; 0.33; 0.07; − 0.41; − 0.07; 1.22; and HD measures (Fig. 3): pair 5–11 (bias) = 0.09; 0.17; 0.13; 0.04; − 0.39; − 0.33; − 0.06. Following Wilcoxon matched-paired signed rank tests revealed no differences in HR and HD between both sessions for pair 5–10 (all p > 0.05; Supplementary Table S7) and only one significant difference in HR for pair 11 (p = 0.004). However, as we did not correct for multiple testing (in order to detect subtle differences), the difference for pair 11 (mean HR = 4.4 vs 3.1) was still acceptable.

Hence, all 7 additional odor pairs showed acceptable agreement between test–retest sessions. The next step included calculations of normative values for HR and HD.

### Normative values for HR and HD for evaluated odor pairs

In accordance to established test methods in chemosensory research, we calculated normative values (e.g., mean, standard deviation, percentiles) for interpretation of HR and HD^29, 30, 46, 47^. We only included results from normosmic subjects of the olfactory reference group aged between 18 and 35 years, since young adults demonstrate the best olfactory test results in quantitative terms^32^. Similar to above mentioned test methods in chemosensory research, we also defined the 10th percentile as cut-off value to distinguish between “normal” or “reduced/negative” HR and HD. We first calculated normative values of HR and HD for (i) each odor pair separately, (ii) the short version of SSParoT based on HR and HD from odor pair 1 to 4 (representing odors from the 16-item Sniffin’ Sticks Identification test), and (iii) the extended version of SSParoT based on HR and HD from all odor pairs (1 to 11). SSParoT results (HR and HD) can therefore be interpreted for each odor pair separately, or in comparison to cut-off scores of the short or extended SSParoT version depending on available felt-tip pens (i.e. interpretation of results based on cut-off scores of the short version of SSParoT when only using the 16-item Sniffin’ Sticks Identification test). Furthermore, since the hedonic judgement of odors shows sex-specific differences, all normative data are stratified by sex (Tables 3 and 4).

**Table 3:** Normative data stratified by sex for hedonic range (HR) of (i) the short version of SSParoT (including results from odor pair 1 to 4 using the 16-item Sniffin’ Sticks Identification test), (ii) the extended version of SSParoT (including results from pair 1 to 11), and (iii) each odor pair separately.

	Mean (standard deviation)	Min to max	10th percentile
**Short version**			
Male	4.5 (2.3)	− 3.0 to 8.0	1.0
Female	4.5 (2.0)	− 2.0 to 7.0	2.0
**Extended version**			
Male	4.1 (2.3)	− 7.0 to 8.0	1.0
Female	4.4 (2.1)	− 6.0 to 8.0	2.0
**Pair 1**			
Male	5.6 (1.9)	2.0 to 8.0	2.0
Female	5.5 (1.3)	3.0 to 7.0	3.0
**Pair 2**			
Male	4.9 (2.2)	− 1.0 to 7.0	1.4
Female	4.6 (1.9)	2.0 to 7.0	2.0
**Pair 3**			
Male	3.8 (2.5)	− 3.0 to 8.0	0.0
Female	3.5 (2.1)	− 2.0 to 6.0	0.0
**Pair 4**			
Male	3.8 (2.0)	0.0 to 7.0	1.0
Female	2.1 (2.1)	0.0 to 7.0	0.0
**Pair 5**			
Male	4.0 (3.0)	− 7.0 to 8.0	1.0
Female	5.0 (1.3)	3.0 to 8.0	4.0
**Pair 6**			
Male	4.2 (2.1)	− 2.0 to 7.0	2.0
Female	4.0 (2.9)	− 6.0 to 8.0	0.0
**Pair 7**			
Male	3.7 (1.7)	0.0 to 7.0	1.0
Female	4.2 (2.2)	− 2.0 to 7.0	1.2
**Pair 8**			
Male	3.1 (2.8)	− 3.0 to 7.0	− 1.0
Female	3.9 (2.6)	− 5.0 to 8.0	0.8
**Pair 9**			
Male	4.1 (2.0)	0.0 to 7.0	1.0
Female	4.7 (1.9)	1.0 to 7.0	1.8
**Pair 10**			
Male	3.5 (2.5)	− 3.0 to 7.0	0.0
Female	4.3 (2.4)	− 4.0 to 7.0	0.8
**Pair 11**			
Male	4.8 (1.7)	1.0 to 8.0	2.0
Female	4.7 (1.3)	1.0 to 7.0	3.0

Results were derived from subjects between 18 and 35 years (pair 1 to 4, n = 50, 31 males, 19 females; pair 5 to 11, n = 56, 29 males, 27 females).

**Table 4:** Normative data stratified by sex for hedonic direction (HD) of (i) the short version of SSParoT (including results from odor pair 1 to 4 using the 16-item Sniffin’ Sticks Identification test), (ii) the extended version of SSParoT (including results from pair 1 to 11), and (iii) each odor pair separately.

	Mean (standard deviation)	Min to Max	10th percentile
**Short version**			
Male	− 0.1 (1.5)	− 2.5 to 3.5	− 2.0
Female	− 0.1 (1.6)	− 3.0 to 3.5	− 2.0
**Extended version**			
Male	− 0.1 (1.2)	− 2.5 to 3.5	− 1.5
Female	− 0.1 (1.2)	− 3.0 to 3.5	− 2.0
**Pair 1**			
Male	− 1.3 (0.9)	− 2.0 to 1.0	− 2.0
Female	− 1.4 (0.7)	− 2.0 to − 0.5	− 2.0
**Pair 2**			
Male	− 0.6 (1.0)	− 2.5 to 2.5	− 1.5
Female	− 0.9 (1.0)	− 3.0 to 0.5	− 2.5
**Pair 3**			
Male	0.2 (1.1)	− 1.5 to 2.5	− 1.0
Female	0.0 (1.1)	− 2.0 to 2.0	− 2.0
**Pair 4**			
Male	1.4 (1.4)	− 1.5 to 3.5	− 0.5
Female	2.0 (1.0)	0.0 to 3.5	0.5
**Pair 5**			
Male	0.5 (0.9)	− 1.0 to 2.5	− 0.5
Female	− 0.6 (0.7)	− 1.0 to 1.5	− 0.6
**Pair 6**			
Male	0.2 (1.1)	− 2.0 to 2.5	− 1.0
Female	− 0.6 (0.6)	− 1.0 to 1.5	− 0.6
**Pair 7**			
Male	0.0 (1.1)	− 2.5 to 1.5	− 1.5
Female	− 1.2 (1.0)	− 2.0 to 2.0	− 1.2
**Pair 8**			
Male	− 0.2 (1.0)	− 2.5 to 1.5	− 1.5
Female	− 1.5 (1.1)	− 1.5 to 3.5	− 1.5
**Pair 9**			
Male	− 0.1 (1.1)	− 2.5 to 1.5	− 1.5
Female	− 2.0 (1.0)	− 2.5 to 1.5	− 2.0
**Pair 10**			
Male	− 0.3 (0.9)	− 2.0 to 1.5	− 1.5
Female	− 2.0 (1.0)	− 2.5 to 1.5	− 2.0
**Pair 11**			
Male	− 0.6 (0.7)	− 1.5 to 1.0	− 1.5
Female	− 1.6 (0.8)	− 2.0 to 1.5	− 1.6

Results were derived from subjects between 18 and 35 years (pair 1 to 4, n = 50, 31 males, 19 females; pair 5 to 11, n = 56, 29 males, 27 females).

## Discussion

Significant progress has been made in the objective diagnosis of quantitative olfactory dysfunction, but there is a gap in tests for qualitative impairments^7, 34–39, 48, 49^. Although distorted odor perceptions have long been a well-known symptom of qualitative OD, little is known about the pathophysiology, clinical course, and potential prognostic value^9, 10, 22, 50–52^. For these reasons, we established the SSParoT, which is based on the presentation of hedonically oppositely valued odors using felt-tip pens^29, 30^. SSParoT measures hedonic range (HR) and hedonic direction (HD) of these odor pairs, which can then be interpreted individually (for each pair) compared to normative data based on the 10^th^ percentile. Here we showed that 4 pairs (8 odors) from the original Sniffin’ Sticks Identification test are suitable for hedonic testing. In addition, the reproducibility of SSParoT was also validated, as test–retest results showed substantial agreement. Moreover, 24 additional odors were introduced from which 14 (7 pairs) also appeared to be valid for hedonic testing. Finally, results from these 14 additional odors have also demonstrated reproducibility.

In reference to the development process of SSParoT, the first aim of this work was to define suitable, objectifiable parameters that are exemplary for qualitative OD. Since previous works provided evidence that parosmia is usually characterized as hedonically unpleasant, evaluation of hedonic estimates seemed intuitive. Measurements of perceived pleasantness of different odors were achieved by using 9-point hedonic scales with visual representation, which have been long established in chemical sensory science^53, 54^. We defined HR as the perceivable hedonic distance (dynamic range) between two oppositely valenced odors, while HD serves as an indicator for the general hedonic direction (pleasant or unpleasant) of odors in daily life.

The second objective of our study was to develop a test, that is readily available, practical in daily use, and bears the potential of reusability. Based on the 16-item Sniffin’ Sticks Identification test, part of the SSParoT can be immediately implemented into clinical routine by using pre-exising tools based on a different test protocol ^7, 29, 30, 55^. Long shelf-life also ensures that this new method is cost-effective^29, 30^. To minimize the potential bias of habitutation and adaptation processes (repeated presentation of the same odors), SSParoT should be performed based on the proposed protocol prior to odor identification testing^56, 57^.

The third objective of this study was to develop a method, which controls for the association between hedonic judgement and perceived intensity, since previous studies have demonstrated a close interrelation^34, 58–60^. We therefore adapted intensity (concentration) of 24 additional odors from experiment three based on results from the first experiment using the weakest and most intense odors as benchmarks. Intensity ratings confirmed preliminary experiments, showing that intensity ratings were comparable to the benchmark (first experiment).

Regarding the normative data presented in this study, these were derived from healthy volunteers of the olfactory reference group, who yielded olfactory test scores in the normosmic range^32^. Previous published test methods in chemosensory research that measure quantitative olfactory and gustatory function provided evidence for the usefulness of normative data (and associated cut-off scores) in the clinical evaluation of patients with chemosensory dysfunctions and for research purposes^30, 31, 61^. Regarding quantitative olfactory function, the 10th percentile (based on normative data derived from healthy subjects of the olfactory reference group) has been proposed as “general” cut-off score to distinguish between healthy and diseased^31^. The authors reasonably noted that the interpretation of test results based on this cut-off score remains to some extend an “arbitrary” decision, since it was derived from subjects of the age group with the overall best olfactory function. Since olfactory function deteriorates with age ^31–33^, individual test results must be always interpreted within each age group. Based on these considerations, we propose that the 10th percentile in HR and HD for each odor pair might also serve as “general” cut-off score to distinguish between “normal” and “reduced/impaired” hedonic perception in terms of qualitative olfactory function. Based on the main complaints of patients with qualitative OD (e.g., unpleasant hedonic perceptions), we would expect those patients with odor-specific parosmia to achieve odor-pair specific HR and HD below the 10th percentile, while those with non-specific parosmia triggered by any odor to achieve HR and HD below the 10th percentile in the short (odor pair 1 to 4) and extended (odor pair 1 to 11) version of SSParoT. For patients with phantosmia, we would expect similar results compared to those with non-specific parosmia. However, since unpleasant hedonic perceptions have been historically more commonly associated with parosmia—it has even been termed ‘cacosmia’^8^—HD (representing general hedonic experiences of odors in daily life) might be lower in patients with parosmia compared to those with phantosmia. Since HR and HD are based on the same parameters, while addressing different aspects of hedonic perception, both high and low HR can result in a value of zero in HD.

A previous study using a method similar to that of SSParoT which assessed HR in patients with Parkinson's disease (PD) revealed that PD patients with concurrent smell loss exhibit significantly lower HR compared to healthy, normosmic subjects. This difference was also found in normosmic PD patients compared to healthy, normosmic subjects. The authors hypothesized that this difference might be mediated independently from quantitative olfactory dysfunction, hence reduced HR (anhedonia) might be a distinct olfactory symptom in PD patients. Moreover, the authors also showed that HR was negatively correlated with the Snaith–Hamilton–Pleasure-Scale, which measures self-perceived anhedonia (higher scores indicated higher levels of present state of anhedonia). This finding further adds evidence to the practical framework and validity of HR, since higher self-perceived state of anhedonia was also associated with lower measured HR^62^. Furthermore, preliminary results from three patients with qualitative OD included in the current investigation—two patients with non-specific parosmia and one patient with phantosmia (Supplementary Table S8) provided further evidence for the proof of concept of SSParoT. Compared with normative data, all patients performed below the 10th percentile in HR based on the extended version of SSParoT.

This test method is unique as it partly uses established tests for olfactory performance to study the effect of odor hedonic perception. However, this study also has limitations. The first and main limitation remains the full validation of SSParoT, since we only included three patients with qualitative OD. Additionally, the usefulness of both parameters (i.e. HR and HD) and cut-off scores of the short and extended versions of SSParoT also need further investigation including larger cohorts of patients with qualitative OD. Secondly, the effect of cross-adaptation during odor presentation^41, 63–65^ (i.e., the presentation of one odor raises olfactory threshold and decreases the perceived intensity to another odor) during repeated judgements within a test session might have biased the results of odor intensity and hedonic valence ratings. However, since we used an interstimulus interval of 30 s between odor presentations^56, 57^, cross-adaptation might not have biased our results to a large extent. Thirdly, the general labeled magnitude scale (gLMS)^66, 67^ might have been an appropriate alternative for the assessment of perceptual responses compared to the nine-point hedonic scale and the visual analogue scale for intensity estimates. Fourthly, although we provided first normative data, we only included subjects from the olfactory reference age group younger than 35 years. A previous study on hedonic responses to various odors in different age groups provided evidence that these responses might be mediated by odor semantic knowledge that differs during the course of life^68^. Therefore, further normative data are needed for different age groups to allow an individual interpretation of results.

This study adds to the current literature on olfactory test methods in three important ways. First, it introduces a new method that measures hedonic estimates of odors (HR and HD, respectively) based on pre-existing tools, allowing clinicians to integrate this new test into clinical routine immediately. Second, it provides evidence that fourteen additional odors are also suitable for hedonic testing. Third, it provides the first normative data derived from healthy, normosmic subjects of the olfactory reference group for the interpretation of test results.

## Methods

### Ethics approval

The study was approved by ethic committees at the University Erlangen-Nürnberg (EK: 4486 and 222_12) and the Medical University of Vienna (EK: 2010/1144). The study was conducted according to the Declaration of Helsinki on biomedical research involving human subjects. Written informed consent was obtained from all subjects prior to the participation.

### Study population

This multicenter, prospective experimental study was conducted at the Medical University of Vienna and University of Erlangen–Nürnberg. Healthy adults were recruited through invitational notices displayed at various pin boards at all study sites. Eligible subjects met the inclusion criteria of no self-reported complaints (quantitative and qualitative) regarding the senses of smell and taste. All participant underwent a routine ear-, nose-, and throat examination and a medical history was obtained. A total of 179 subjects were screened and 162 normsomic subjects (89 male, 74 females, mean ± SD age, 33.5 ± 15.6 years, range 18–82) were enrolled over two study centers. In addition, three patients presenting with self-reported qualitative olfactory dysfunction (parosmia and phantosmia) were recruited at the University of Erlangen-Nürnberg (1 male, 2 females, mean ± SD age, 31.3 ± 12.5 years, range 18–48).

### Development of the SSParoT

Hedonic estimates were assessed using a 9-point hedonic scale ^53, 54^ (Supplementary Fig. S1). This scale has first been introduced by Peryam et al.^53^ to measure food preferences and has quickly been adopted by the industry to measure the acceptability of various products related to food and cosmetics. We hypothesized that hedonic estimates of odors can be categorized into (i) pleasant, (ii) neutral, and (iii) unpleasant^69^. We therefore defined hedonic range (HR) as the perceivable hedonic distance between two oppositely valenced odors, and hedonic direction (HD) as an indicator for general hedonic experiences of odors in daily life (e.g., how pleasant will a pleasant odor be perceived and how unpleasant will an unpleasant odor be perceived). Both scales were developed based on the practical framework that patients with qualitative OD mainly complain about unpleasant hedonic perceptions^7–10^. A predefined odor pair consisting of one pleasant (E1) and one unpleasant (E2) odor were therefore presented pairwise and subjects’ task was to rate the hedonic tone. Both scores were then calculated as follow (for each pair separately):

(i) Hedonic range of each odor pair was defined as the difference between both estimates (in whole numbers):
  $$HR=E1-E2$$
  Given that the hedonic scale ranges from -4 to + 4, the newly defined HR can take whole numbers ranging from -8 to 8.
(ii) Hedonic direction of each odor pair was defined as the mean value between both estimates (in whole numbers):
  $$HD=\frac{E1+E2}{2}$$

Since the hedonic scale ranges from - 4 to + 4, HD can take whole or half numbers ranging from − 4 to + 4.

Felt-tip pens (Burghart Messtechnik GmbH, Wedel, Germany) were chosen to serve as the carrier medium for all odors . These felt tip pens are widely used and characterized by (i) reusability, (ii) long shelf-life, (iii) easy application, and (iv) the possibility to be self-filled using blank pens^29, 30^.

In four experiments, we assessed (i) the suitability of the 16-item Sniffin’ Sticks Identification test to assess hedonic estimates, (ii) the reliability of HR and HD of odor pairs selected from the 16-item Sniffin’ Sticks Identification test, (iii) the suitability of 24 additional odors for hedonic estimates, and (iv) the reliability of HR and HD of odor pairs selected from the extended version. Since perceived intensity of odors is known to be associated with pleasantness^34, 58–60^, we simultaneously evaluated intensity of each presented odor on a visual analogue scale ranging from 0 to 10 (left hand end: 0 = no intensity, right hand end: 10 = strong intensity).

### Experiment 1: subjects

Fifty-two subjects with no complaints regarding the sense of smell were screened using the German-version of the 16-item Sniffin’ Sticks Identification test and 50 normosmic subjects (28 males, 22 females, mean ± SD age, 28.8 ± 9.8 years, range 18–62) from both study centers (Erlangen, n = 20; Vienna, n = 30) were included during one visit.

### Experiment 1: design

This experiment was carried out at the Friedrich-Alexander University Erlangen-Nürnberg and the Medical University of Vienna. The aim was to assess the hedonic estimates of commonly used odors from the original 16-item Sniffin’ Sticks test and to identify hedonically oppositely valenced odors (pleasant and unpleasant). Odors were presented according to the order of the original 16-item Identification test with a break of at least 30 s in-between each odor to prevent the effect of olfactory desensitization^44, 56, 57^. Subjects’ task was to rate hedonic estimates, followed by intensity ratings. At the end of the first experiment, subjects underwent olfactory testing using the 16-item Identification test. Normal olfactory function was defined as ≥ 11 out of 16 possible points^32^.

### Experiment 1: statistical analysis

Hedonic estimates were first grouped into clusters based on hierarchical clustering using the squared Euclidean distance metric and average-linkage method. Based on visual inspection of the cluster profiles and the inverse scree test (elbow) method^70^, we determined the optimal number of clusters to be three. Cluster membership of odors was then identified in a second step using the non-hierarchical K-means analysis and clusters were named according to their hedonic valence: Cluster 1 = pleasant odors, Cluster 2 = neutral odors, and Cluster 3 = unpleasant odors. Subsequently, HR and HD of paired odors (pleasant and unpleasant) from Cluster 1 and 3 were calculated based two different methods (i) pairing the most pleasant odor from Cluster 1 with the most unpleasant odor from Cluster 3 and (ii) pairing the most pleasant odor from Cluster 1 with the least unpleasant odor from Cluster 3. In a final step, HR of each method were tested for significant differences using Kruskal–Wallis tests in order to pick out the pairing method with the smallest difference.

### Experiment 2: subjects

This second experiment screened 37 subjects and included 33 normosmic subjects for further analysis (11 males, 22 females, mean ± SD age, 36 ± 19 years, range 22–80). These subjects visited the study centers twice with at least one day separation between visits (mean ± SD duration, 11.8 ± 7.3 days, range 2–34).

### Experiment 2: design

This second experiment took place at the Friedrich-Alexander University Erlangen-Nürnberg and was designed to assess the reliability of HR and HD estimates. Hedonically matching pairs of opposing odors selected from experiment 1 were presented pairwise with a break of 30 s in-between odors. Odor pairs were presented starting with the most pleasant odor (counterbalanced), followed by its matched odor pair: Pair 4: Banana and Clove, pair 3: Pineapple and Turpentine, pair 2: Apple and Garlic, pair 1: Peppermint and Fish. Subjects’ task was to estimate hedonic valence and intensity as described in experiment 1. Olfactory performance was screened using a randomized procedure after the first or second visit to detect potential confounders based on above described cut-off values.

### Experiment 2: statistical analysis

HR and HD were calculated for each pair and session. To assess agreement between measurements, we first calculated the absolute difference (bias) in HR and HD scores for each pair of odors, followed by graphical visualization using Bland–Altman plots^71^. In a next step, we compared HR and HD of both sessions using Wilcoxon matched-paired signed rank tests without correcting for multiple comparisons in order to identify small differences between both sessions.

### Experiment 3: subjects

The third part of the experiment screened another 56 subjects and included 52 nomosmic subjects (25 males, 27 females, mean ± SD age, 30.7 ± 13.8 years, range,19–75) from both study centers (Erlangen, n = 22; Vienna, n = 30) during one visit.

### Experiment 3: design

This experiment was carried out at the Friedrich-Alexander University Erlangen–Nürnberg and the Medical University of Vienna. Twenty-four additional odors (Frey und Lau, Henstedt-Ulzburg, Germany; Supplementary Table S1) were diluted in dipropylen-glycol (Merck 803265) and 4 mL were filled in felt-tip pens (Burghart Messtechnik GmbH, Wedel, Germany). The concentrations of odors were adjusted in a preliminary experiment carried out by the authors using intensity ratings from experiment 1 as benchmark: Fish (most intense) and Anise (least intense). Odor concentrations and the order of odor presentation are summarized in Supplementary Table S1. Olfactory performance was screened after the hedonic and intensity estimates.

### Experiment 3: statistical analysis

Hedonic estimates were clustered following the same methods described in experiment 1. Again, three clusters were determined as the optimal number: Cluster 1 = pleasant, Cluster 2 = neutral, and Cluster 3 = unpleasant. Subsequently, pleasant and unpleasant odors from Cluster 1 and 3 were paired using the same method as described in experiment 1. Finally, HR of each newly matched pair were calculated and tested for significant differences using the Kruskal–Wallis test to validate the odor pairs.

### Experiment 4: subjects

The last experiment screened another 34 subjects and included a total of 27 normosmic subjects (12 males, 15 females, mean ± SD age, 43 ± 19 years, range 20–82) for further analysis. Subjects visited the study center twice with at least one day separation between visits (mean ± SD duration, 13.5 ± 16.3 days, range, 1–56).

### Experiment 4: design

This experiment was conducted at the Friedrich-Alexander University Erlangen-Nürnberg. As has been the case in experiment 2, the last experiment was also designed to investigate the reliability of HR and HD estimates of the additionally matched pairs of opposing odors. Odor pairs were presented starting with the most pleasant odor, followed by its matched odor pair (counterbalanced): Pair 5: Peach and Butter; pair 6: Coco and n-Butyric acid; pair 7: Caramel and iso-Butyric acid; pair 8: Raspberry and Indole; pair 9: Ice bonbon and Skatole; pair 10: Lemon and Civet; pair 11: Orange and Valeric acid. Olfactory performance was either tested after the first or second visit based on a study protocol inherent randomization.

### Experiment 4: statistical analysis

HR and HD were calculated for each of the newly added odor pairs. Bland–Altman plots were visualized, following calculations of the absolute agreement between visits. Group comparisons were performed using Wilcoxon matched-paired signed rank tests to assess differences for HR and HD between both visits. In order to detect subtle differences, we did not correct for multiple testing.

### Normative values

We only included HR and HD results from normosmic subjects of the olfactory reference group aged between 18 and 35 years: Odor pair 1 to 4 (n = 50, experiment 1 and experiment 2-first visit) and odor pair 5 to 11 (n = 56, experiment 3 and experiment 4-first visit). Descriptive statistics of HR and HD (i.e., mean, standard deviation, min–max, 10th percentile) were calculated for (i) each odor pair separately, (ii) the short version of SSParoT, based on HR and HD of odor pair 1 to 4, and (iii) the extended version of SSParoT based on HR and HD of odor pair 1 to 11.

### Statistical analysis

Statistical analysis and data visualization were performed using SPSS 25.0 (Chicago, IL, USA) and GraphPad Prism 8.4.2 (GraphPad Software, Inc., La Jolla, CA). Normality of data was assessed based on histograms and Q-Q plots. Alpha level was set at 0.05.

## Supplementary information

Supplementary Information.

## Data Availability

The institutional ethics committee (Ethikkommission der Medizinischen Universität Wien—Borschkegasse 8b/E06, 1090 Vienna) is imposing legal and ethical restrictions on the present data. Requests for data will be administered by the corresponding author.
