# Supplementary material for: Assessment of odor hedonic perception: the Sniffin’ sticks parosmia test (SSParoT)
Source: Sci Rep. 2020 Oct 22;10:18019. doi: 10.1038/s41598-020-74967-0 (PMC7581750; doi:10.1038/s41598-020-74967-0)

**Supplementary material to:**

**Assessment of Odor Hedonic Perception: The Sniffin` Sticks Parosmia Test (SSParoT)**

David T. Liu^1^, Antje Welge-Lüssen^2^, Gerold Besser^1^, Christian A. Mueller^1 +^, Bertold Renner^3,4^

^1^ Department of Otorhinolaryngology, Head and Neck Surgery, Medical University of Vienna, Vienna, Austria

^2^Department of Otorhinolaryngology, University Hospital, University of Basel, Basel, Switzerland

^3^Institute of Experimental and Clinical Pharmacology and Toxicology, Friedrich-Alexander Universität Erlangen-Nürnberg, Erlangen, Germany

^4^Institute of Clinical Pharmacology, Medical Faculty Carl Gustav Carus, Technische Universität Dresden, Dresden, Germany

+ corresponding author, christian.a.mueller@meduniwien.ac.at

**Tables**

**Supplementary Table 1.** Cluster membership of K-means clustering of the 16-odors used in experiment 1.

| Odor | Cluster | Distance |
| --- | --- | --- |
| Orange | 1 | 11.877 |
| Shoe leather | 1 | 9.715 |
| Lemon | 1 | 10.788 |
| Liquorice | 1 | 7.878 |
| Coffee | 1 | 12.964 |
| Anise | 1 | 8.469 |
| Turpentine | 2 | 8.089 |
| Garlic | 2 | 7.512 |
| Clove | 2 | 10.195 |
| Fish | 2 | 9.134 |
| Cinnamon | 3 | 14.583 |
| Peppermint | 3 | 11.121 |
| Banana | 3 | 9.522 |
| Apple | 3 | 8.641 |
| Pineapple | 3 | 8.327 |
| Rose | 3 | 13.304 |

**Supplementary Table 2.** Results of odor pairing using method one (most pleasant odor from Cluster 1 matched with the least unpleasant odor from Cluster 3) and method two (most pleasant odor from Cluster 1 matched with the most unpleasant odor from Cluster 3) from experiment 1 (n=50). Abbreviations: HR= hedonic range, HD= hedonic direction, SD= standard deviation.

|  |  |  |  | | | |
| --- | --- | --- | --- | --- | --- | --- |
|  | Odor 1 | Odor 2 | HR | | HD | |
| Pairs |  |  | Mean (SD) | Range | Mean (SD) | Range |
| Method one | | | | | | |
| 1 | Peppermint | Fish | 5.2 (1.9) | 0to 8 | -1.9 (0.3) | -2to -0.5 |
| 2 | Apple | Garlic | 5.2 (2.0) | -1to 8 | -0.8 (0.9) | -3.5to 1.5 |
| 3 | Pineapple | Turpentine | 4.4 (2.1) | -2to 7 | 0.1 (1) | -3to 2.5 |
| 4 | Banana | Clove | 4.4 (1.9) | -2to 7 | 2.2 (1) | -1to 3.5 |
| Method two | | | | | | |
| 1 | Peppermint | Clove | 3.3 (2.2) | -1to 7 | -0.2 (1.3) | -3.5to 3 |
| 2 | Apple | Turpentine | 4.0 (2.3) | -4to 7 | -0.2 (1.1) | -3.5to 2.5 |
| 3 | Pineapple | Garlic | 5.7 (1.8) | 1to 8 | -0.6 (0.8) | -3to 0.5 |
| 4 | Banana | Fish | 6.2 (1.6) | 1to 8 | -0.6 (0.71) | -3to 1 |

**Supplementary Table 3.** Test- retest results of hedonic range (HR) and hedonic direction (HD) of odor pairs 1-4 selected from the 16-item Sniffin’ Sticks test (n=33). Abbreviations: HR= hedonic range, HD= hedonic direction, SD= standard deviation.

|  |  |  | Visit 1 | | | | Visit 2 | | | |  | |
| --- | --- | --- | --- | --- | --- | --- | --- | --- | --- | --- | --- | --- |
|  | Odor 1 | Odor 2 | HR | | HD | | HR | | HD | | P value | |
| Pairs |  |  | Mean (SD) | Range | Mean (SD) | Range | Mean (SD) | Range | Mean (SD) | Range | HR | HD |
| 1 | Peppermint | Fish | 6.0 (1.8) | 2 to 8 | -0.4 (0.8) | -3 to 1 | 6.3 (1.2) | 4 to 8 | -0.3 (0.6) | -1 to 8 | 0.7 | 0.8 |
| 2 | Apple | Garlic | 5.0 (1.8) | -6 to 8 | -0.4(1.0) | -2.5 to 2.5 | 5.3 (3.2) | -1.5 to 3.5 | -0.3 (1.1) | -0.5 to 4 | 0.2 | 0.6 |
| 3 | Pineapple | Turpentine | 3.0 (3.1) | -2 to 3 | 0.5 (1.6) | -2 to 3 | 3.1 (2.8) | -3 to 8 | 0.3 (1.3) | -2.5 to 3.5 | 0.9 | 0.4 |
| 4 | Banana | Clove | 3.2 (2.4) | 0 to 7 | 0.5(1.3) | -2 to 2 | 2.7 (2.5) | -3 to 8 | 0.8 (1.3) | -3 to 1 | 0.6 | 0.4 |

**Supplementary Table 4.** Odors and concentrations used for experiment 1 and 3 with corresponding odor numbers (Frey und Lau, Henstedt-Ulzburg, Germany). Volume per stick 4 ml, used solvent dipropylene glycol (Merck 803265).

|  | Experiment 1 | Sniffin’ Sticks | Experiment 3 | Odor number | Concentration in % by volume or weight |
| --- | --- | --- | --- | --- | --- |
| 1 | Anise | #15 | Apple | #P0600954 | 20 |
| 2 | Apple | #11 | Butter | #P0620830 | 20 |
| 3 | Banana | #5 | Caramel | #P0605456 | 20 |
| 4 | Cinnamon | #3 | Civet | #S0100211 | 2.5 |
| 5 | Clove | #12 | Coco | #P0601708 | 20 |
| 6 | Coffee | #10 | Cola | #P0222975 | 10 |
| 7 | Fish | #16 | Ginger | #S0100054 | 5 |
| 8 | Garlic | #9 | Grass | #P0603760 | 20 |
| 9 | Lemon | #6 | Ice bonbon | #P0611334 | 20 |
| 10 | Liquorice | #7 | Indole | #S0400841 | 2.5 |
| 11 | Orange | #1 | iso-Butyric acid | #S0401056 | 5 |
| 12 | Peppermint | #4 | Lavender | #P0123527 | 10 |
| 13 | Pineapple | #13 | Lemon | #S0402085 | 10 |
| 14 | Rose | #14 | Menthol | #S0400621 | 67 |
| 15 | Shoe leather | #2 | n-Butyric acid | #S0400929 | 1 |
| 16 | Turpentine | #8 | Orange | #P0606416 | 20 |
| 17 |  |  | Peach | #P0606040 | 10 |
| 18 |  |  | Pear | #P0603566 | 10 |
| 19 |  |  | Raspberry | #P0602962 | 20 |
| 20 |  |  | Rose | #S0400707 | 20 |
| 21 |  |  | Skatole | #S0400974 | 0.1 |
| 22 |  |  | Strawberry | #P0603875 | 20 |
| 23 |  |  | Valerian | #S0100006 | 10 |
| 24 |  |  | Valeric acid | #S0401225 | 5 |

**Supplementary Table 5.** Cluster membership of K-means clustering of the 24-odors used in experiment 3.

| Odor | Cluster | Distance |
| --- | --- | --- |
| Valerian | 1 | 11.430 |
| Lavender | 1 | 12.241 |
| Ginger | 1 | 10.442 |
| Grass | 1 | 10.538 |
| Rose | 1 | 12.257 |
| n-Butyric acid | 2 | 9.908 |
| Civet | 2 | 9.367 |
| Indole | 2 | 10.324 |
| Skatole | 2 | 7.821 |
| Valeric acid | 2 | 8.419 |
| Iso-Butyric acid | 2 | 7.974 |
| Butter | 2 | 11.863 |
| Pear | 3 | 10.014 |
| Cola | 3 | 10.883 |
| Peach | 3 | 9.854 |
| Raspberry | 3 | 8.762 |
| Coco | 3 | 8.472 |
| Lemon | 3 | 7.468 |
| Strawberry | 3 | 10.170 |
| Ice bonbon | 3 | 8.452 |
| Apple | 3 | 10.341 |
| Caramel | 3 | 10.682 |
| Menthol | 3 | 12.541 |
| Orange | 3 | 7.644 |

**Supplementary Table 6.** Results of odor pairing from experiment 3 (n=52). Abbreviations: HR= hedonic range, HD= hedonic direction, SD= standard deviation, n-Buty= n-Butyric acid, iso-Buty= iso-Butyric acid.

|  |  |  |  | | | |
| --- | --- | --- | --- | --- | --- | --- |
|  | Odor 1 | Odor 2 | HR | | HD | |
| Pair Number |  |  | Mean (SD) | Range | Mean (SD) | Range |
| 5 | Peach | Butter | 4.4 (2.3) | -2 to 10 | 0.6 (0.8) | -1 to 4 |
| 6 | Coco | n-Buty | 4.4 (2.0) | -1 to 9 | 0.5 (0.9) | -1 to 4 |
| 7 | Caramel | Iso-Buty | 3.9 (2.1) | -2 to 9 | -0.2(1.1) | -2.5 to 5 |
| 8 | Raspberry | Indole | 4.0 (2.5) | -3 to 11 | 0 (1.1) | -2.5 to 6 |
| 9 | Ice bonbon | Skatole | 4.5 (1.9) | 0 to 8 | -0.3 (1.0) | -2.5 to 4 |
| 10 | Lemon | Civet | 4.4 (1.9) | -2 to 9 | -0.3 (0.9) | -2 to 3.5 |
| 11 | Orange | Valeric acid | 4.8 (1.5) | 0 to 8 | -0.5 (0.8) | -2 to 4 |

**Supplementary Table 7.** Test- retest results of hedonic range (HR) and hedonic direction (HD) of odor pairs 5-11 selected from additional 24 odors (n=27). Abbreviations: HR= hedonic range, HD= hedonic direction, SD= standard deviation, n-Buty= n-Butyric acid, iso-Buty= iso-Butyric acid.

|  |  | | Visit 1 | | | | Visit 2 | | | | | |
| --- | --- | --- | --- | --- | --- | --- | --- | --- | --- | --- | --- | --- |
|  | Odor 1 | Odor 2 | HR | | HD | | HR | | HD | | P value | |
| Pair  Number |  |  | Mean (SD) | Range | Mean (SD) | Range | Mean (SD) | Range | Mean (SD) | Range | HR | HD |
| 5 | Peach | Butter | 3.9 (2.8) | -7 to 7 | -0.4 (1.1) | -2 to 2.5 | 4.0 (1.9) | -2 to 7 | 0.3 (1.7) | -1.5 to 2 | 0.4 | 0.6 |
| 6 | Coco | n-Buty | 3.3 (2.6) | -2 to 8 | -0.4 (1.4) | -3 to 3.5 | 3.3 (2.4) | -2 to 7 | -0.56 (0.9) | -3 to 1 | 0.8 | 0.8 |
| 7 | Caramel | Iso-Buty | 4.1 (1.8) | 1 to 8 | 0.4 (0.9) | -1.5 to 2.5 | 3.8 (1.7) | 0 to 7 | 0.22 (0.9) | -2 to 3 | 0.3 | 0.3 |
| 8 | Raspberry | Indole | 3.8 (2.0) | 0 to 7 | -0.2 (1.0) | -1.5 to 3.5 | 3.9 (2.7) | -1 to 8 | -0.22 (0.9) | -1.5 to 1.5 | 0.9 | 0.7 |
| 9 | Ice bonbon | Skatole | 3.8 (2.9) | -3 to 11 | -0.1 (1.3) | -2.5 to 2.5 | 4.2 (2.3) | 0 to 8 | 0.26 (1.2) | -3 to 2.5 | 0.5 | 0.1 |
| 10 | Lemon | Civet | 2.6 (2.4) | -3 to 10 | -0.1 (0.9) | -2.5 to 1 | 2.7 (2.6) | -3 to 7 | 0.20 (0.8) | -1.5 to 1.5 | 0.9 | 0.1 |
| 11 | Orange | Valeric acid | 4.4 (2.1) | 1 to 7 | -0.4 (0.8) | -2.5 to 0.5 | 3.1 (1.7) | 0 to 6 | -0.4 (1.1) | -2.5 to 2.5 | 0.004 | 0.9 |

**Supplementary Table 8.** Preliminary SSParoT results of three patients with self-reported qualitative olfactory dysfunction. Patients were asked as to whether these qualitative impairments could be triggered by any source (binary outcome: yes or no) and for potential causes. After SSParoT testing, patients underwent comprehensive olfactory testing using the Sniffin’ Sticks Threshold, Discrimination, and Identification test (Burghart Medical Technology, Wedel, Germany). Abbreviations: TDI = Test results of the Sniffin’ Sticks Threshold, Discrimination, and Identification test, HR= hedonic range, HD= hedonic direction. The asterix (*) marks values below the 10^th^ percentile in comparison with normative data.

|  | Patient 1 | | Patient 2 | | Patient 3 | |
| --- | --- | --- | --- | --- | --- | --- |
| Qualitative complaint | Parosmia | | Parosmia | | Phantosmia | |
| Triggering source | Nonspecifically any odor | | Nonspecifically any odor | | Nonspecifically | |
| Gender, Age | Male, 48 years | | Female, 18 years | | Female, 28 years | |
| Olfactory function | Severe hyposmia (TDI: 17.25) | | Severe hyposmia (TDI: 21.5) | | Mild hyposmia (TDI: 30) | |
| Reason for olfactory dysfunction | Post-traumatic | | Post-traumatic | | None | |
| Odor pair | HR | HD | HR | HD | HR | HD |
| 1 | 4.0 | 0.0 | 1.0* | -0.5 | 1.0* | 0.5 |
| 2 | 1.0* | -0.5 | 3.0 | 0.5 | 1.0* | 0.5 |
| 3 | -3.0* | 2.5 | 5.0 | -1.5 | -1.0* | -0.5 |
| 4 | 3.0 | -2.5* | 0.0 | 0.0* | 0.0 | 0.0* |
| 5 | 0.0* | 0.0 | -1.0* | 0.5 | 6.0 | 3.0 |
| 6 | -1.0* | 0.5 | 0.0 | 0.0 | 1.0 | 0.5 |
| 7 | 1.0 | 0.5 | 0.0* | 1.0 | 0.0* | 0.0 |
| 8 | 0.0 | 0.0 | 0.0* | 1.0 | -1.0* | -0.5 |
| 9 | -1.0* | -0.5 | 1.0* | 0.5 | -2.0* | -1.0 |
| 10 | 0.0 | 0.0 | 1.0 | 0.5 | -5.0* | -2.5* |
| 11 | 3.0 | -2.5* | 1.0* | -0.5 | 0.0* | 0.0 |
| Overall version | 0.6* | -0.2 | 1.0* | 0.1 | 0.0* | 0.0 |

**Figures**

**Supplementary Figure 1.** Nine-point hedonic scale.


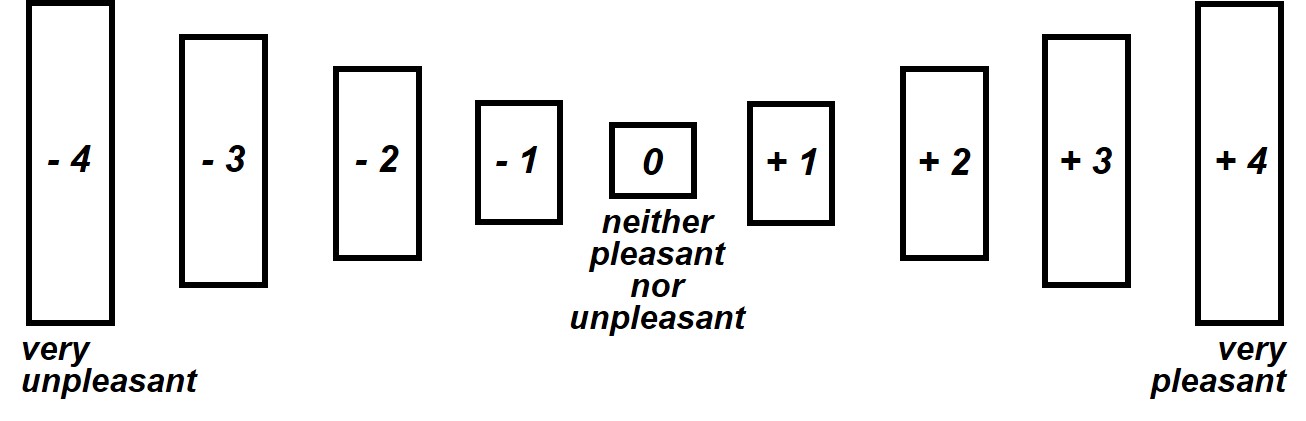


**Supplementary Figure 2**. Dendrogram showing results from hierarchical cluster analysis of hedonic estimates of the 16-item Sniffin’ Sticks Identification test from experiment 1. The horizontal axis represents the distance between clusters.

**
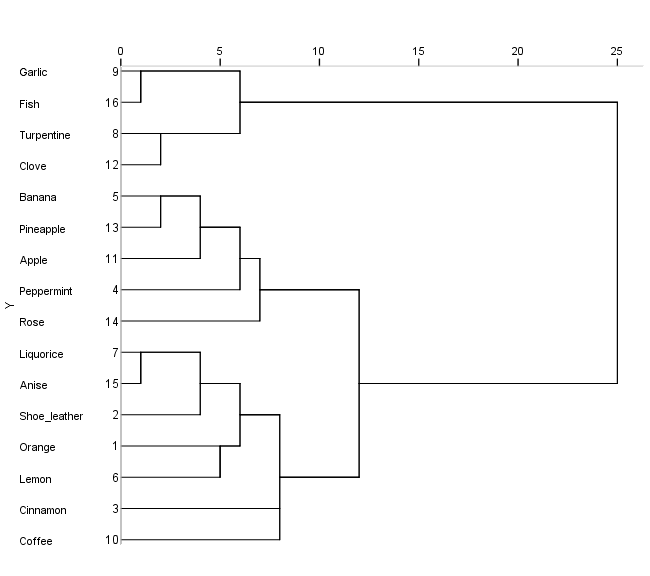
**

**Supplementary Figure 3**. Scree plot showing the agglomeration schedule for hedonic estimates of experiment 1. Arrow shows the point of termination of the clustering process (elbow) after which there is an increase in the coefficient. Based on the plot, the number of clusters revealed is equal to 3 (16–13 = 3).

**
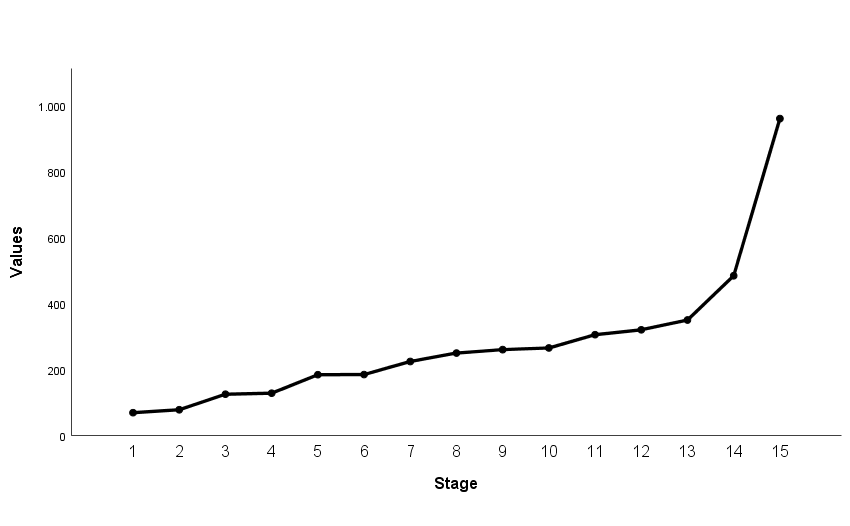
**

**Supplementary Figure 4**. Dendrogram showing results from hierarchical cluster analysis of hedonic estimates of the 24 additional odors from experiment 3. The horizontal axis represents the distance between clusters.


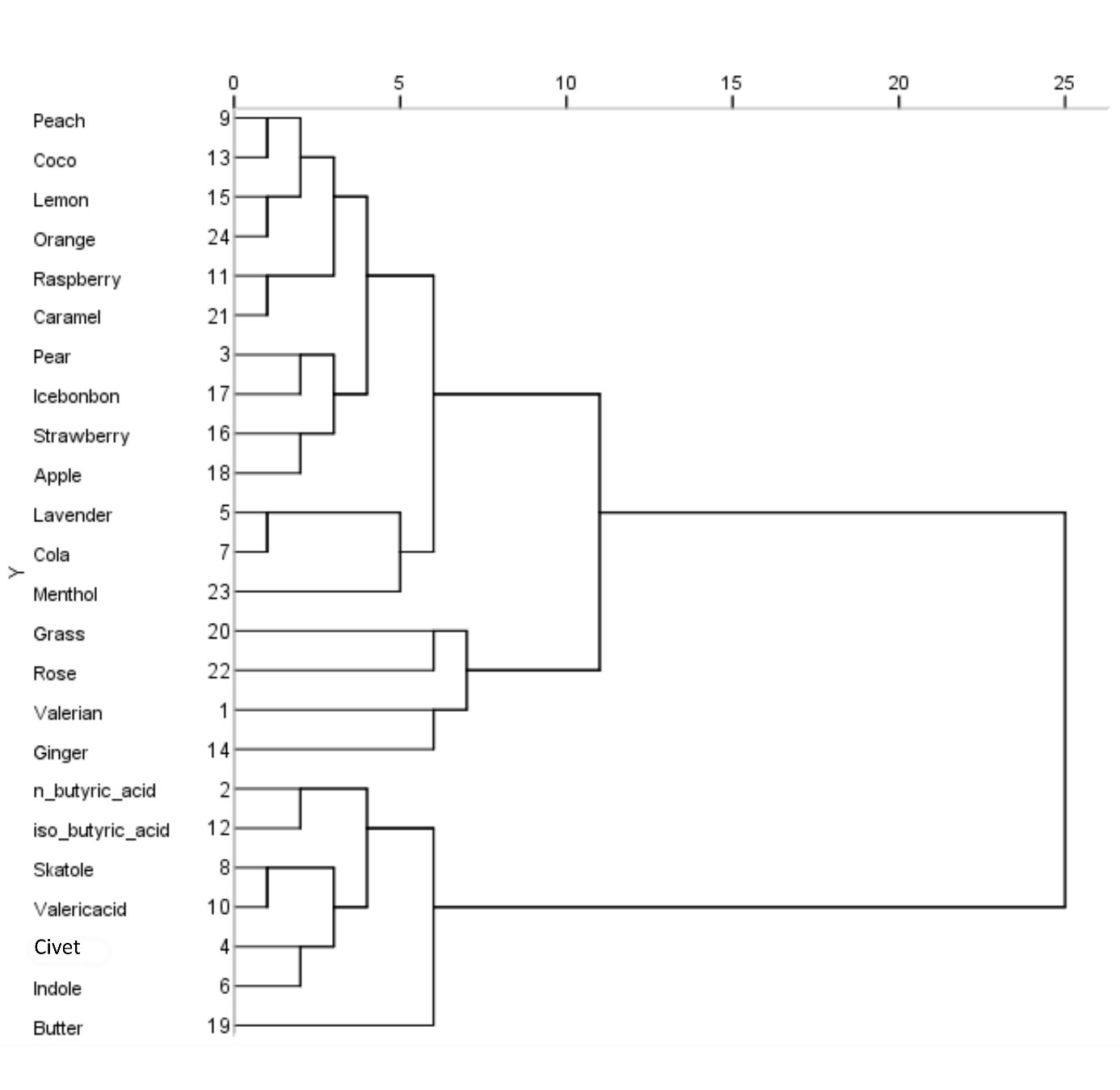


**Supplementary Figure 5**. Scree plot showing the agglomeration schedule for hedonic estimates of experiment 3. Arrow shows the point of termination of the clustering process (elbow) after which there is an increase in the coefficient. Based on the plot, the number of clusters revealed is equal to 3 (23–20 = 3).


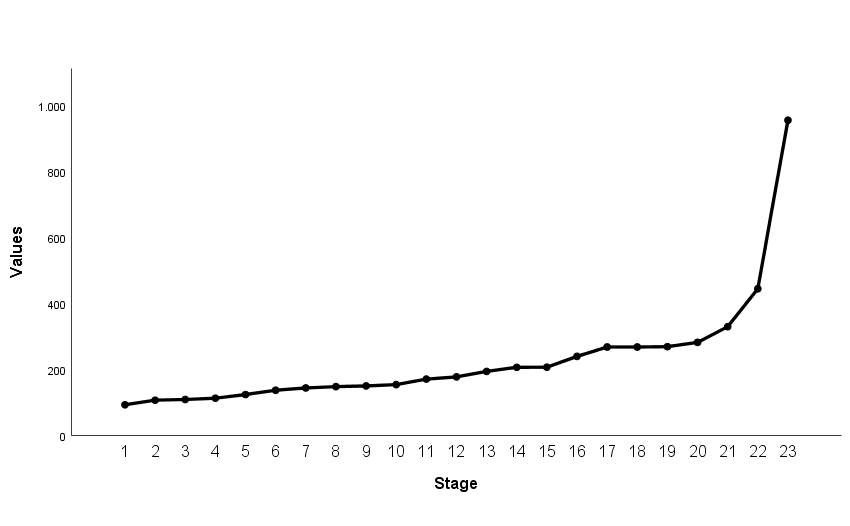

Supplement: Supplementary file 1 — Supplementary Information. [file 41598_2020_74967_MOESM1_ESM.docx]
